# Genetic divergence in soybeans based on phenotypic traits and microsatellite markers

**DOI:** 10.1186/1753-6561-8-S4-P136

**Published:** 2014-10-01

**Authors:** Larissa Barbosa de Sousa, Ana Paula Oliveira Nogueira, Osvaldo Toshiyuki Hamawaki, Ana Carolina Cordeiro Dias, Dão Pedro de Carvalho Neto, Carla Cristina Rodrigues Borges, Valécia Martins de Oliveira, Elequisandra da Costa Araruna, Raphael Lemes Hamawaki

**Affiliations:** 1 Universidade Federal de Uberlândia, ICIAG, Campus Umuarama, Uberlândia-MG, Brazil; 2 Universidade Federal de Uberlândia, INGEB, Uberlândia-MG, Brazil; 3 Universidade Federal de Uberlândia, ICIAG, Uberlândia-MG, Brazil; 4 Universidade Federal de Uberlândia, INBIO, Uberlândia-MG, Brazil

## Background

The success of a breeding program depends on the existence of genetic variability [1]. Therefore, breeders have recommended to the formation of the population-based study, the interbreeding between superior cultivars and divergent, resulting in hybrid combinations of higher heterotic effect, so that in segregating generations it has a greater chance of obtaining transgressive genotypes [2]. Microsatellite markers or Simple Sequence Repeat (SSR) have been widely used to estimate genetic diversity in soybean due to their high degree of polymorphism, co-dominant inheritance, PCR-based detection, allelic diversity and the knowledge of the location of the marker in the genome the species [3]. In this report we present results of agronomic traits and microsatellite markers to evaluate the genetic diversity of 35 soybean genotypes to determine their potential as parents for breeding programs.

## Methods

Thus, we used agronomic traits and microsatellite markers to evaluate the genetic diversity of 35 soybean genotypes to determine their potential as parents for breeding programs. Phenotypic analysis was carried out in the field at Fazenda Capim Branco and molecular analysis was conducted at the Laboratório de Genética do Instituto de Genética e Bioquímica (Genetics Laboratory at the Institute of Genetics and Biochemistry) at the Universidade Federal de Uberlândia (UFU-Federal University of Uberlandia). We evaluated 35 soybean genotypes using seven agronomic traits and nine microsatellites markers. These genotypes were then grouped by UPGMA and Tocher cluster analyses and the relative importance of the agronomic traits was obtained. Molecular analysis was used to calculate polymorphic data for each microsatellite marker. Genetic variability was found for all agronomic traits except for first pod height. UPGMA was used to form ten groups phenotypic analysis and eleven groups for molecular analysis. Additionally, the Tocher method was used to form twelve groups for both of these analyses.

## Results and conclusions

The nine microsatellite markers amplified 26 alleles, ranging from 2 to 4 and averaging 2.88 per marker. Polymorphic data varied between 0.29 and 0.66 and averaged 0.44. Seven potential parents G11, G12, G16, G21, G22, G26 and G33) were identified from the UPGMA and Tocher clusters. These genotypes had average grain yields greater than 5000 kg ha^-1^. Agronomic traits and microsatellite markers are effective tools for the study of genetic diversity in soybeans. Thus, we concluded that using agronomic traits and molecular microsatellite markers together makes it possible to detect potential parents for the soybean breeding program at UFU. Hybrids of the G11, G12, G16, G22, G26 and G33 genotypes promise segregating populations with superior genetic variability.
